# Ambient Air Pollution and Birth Defects in Brisbane, Australia

**DOI:** 10.1371/journal.pone.0005408

**Published:** 2009-04-30

**Authors:** Craig A. Hansen, Adrian G. Barnett, Bin B. Jalaludin, Geoffrey G. Morgan

**Affiliations:** 1 National Center for Environmental Assessment, U. S. Environmental Protection Agency, Durham, North Carolina, United States of America; 2 Institute of Health and Biomedical Innovation & School of Public Health, Queensland University of Technology, Brisbane, Queensland, Australia; 3 Centre for Research, Evidence Management and Surveillance, Sydney South West Area Health Service, Sydney, New South Wales, Australia; 4 School of Public Health and Community Medicine, University of New South Wales, Sydney, New South Wales, Australia; 5 Northern Rivers University Department of Rural Health, University of Sydney, Lismore, New South Wales, Australia; 6 North Coast Area Health Service, Lismore, New South Wales, Australia; Western Illinois University, United States of America

## Abstract

**Background:**

Birth defects are a major public health concern as they are the leading cause of neonatal and infant mortality. Observational studies have linked environmental pollution to adverse birth outcomes, including congenital anomalies. This study examined potential associations between ambient air pollution and congenital heart defects and cleft lip or palate among births in Brisbane, Australia (1998–2004).

**Methods:**

Ambient air pollution levels were averaged over weeks 3–8 of pregnancy among 150,308 births. Using a case–control design, we used conditional logistic regression and matched cases to 5 controls. Analyses were conducted using all births, and then births where the mother resided within 6 and 12 kilometers of an ambient air quality monitor.

**Findings:**

When analyzing all births there was no indication that ambient air pollution in Brisbane was associated with a higher risk of cardiac defects. Among births where the mother resided within 6 kilometers of an ambient air quality monitor, a 5 ppb increase in O_3_ was associated with an increased risk of pulmonary artery and valve defects (OR 2.96, 95% CI: 1.34, 7.52) while a 0.6 ppb increase in SO_2_ was associated with an increased risk of aortic artery and valve defects (OR 10.76, 95% CI: 1.50, 179.8). For oral cleft defects among all births, the only adverse association was between SO_2_ and cleft lip with or without cleft palate (OR 1.27, 95% CI: 1.01, 1.62). However, various significant inverse associations were also found between air pollutants and birth defects.

**Conclusions:**

This study found mixed results and it is difficult to conclude whether ambient air pollution in Brisbane has an adverse association with the birth defects examined. Studies using more detailed estimates of air pollution exposure are needed.

## Introduction

Birth defects are a major public health concern as they are the leading cause of neonatal and infant mortality [1], [2], and a major cause of morbidity later in life. Approximately 14% of neonates are born with a single minor malformation and around 2–3% are born with major malformations. The etiology of congenital malformations are unknown for as many as 60% of all cases, however about 6–8% are associated with exposure to environmental factors, which include teratogenic agents [3].

Observational studies have linked environmental pollution to congenital anomalies [4], with higher risks reported among mothers residing within close proximity to municipal solid waste incinerators [5], [6], landfill sites [7]–[10], and hazardous waste sites [11]–[13]. The main pollutants emitted from these sources are dioxins, which are a group of toxic chemicals that share a similar chemical structure and a common mechanism of toxic action. Dioxins have been characterized as likely human carcinogens and teratogens [14].

Other anthropogenic environmental contaminants include air pollutants such as particulate matter (PM), nitrogen dioxide (NO_2_), sulfur dioxide (SO_2_), carbon monoxide (CO), and the secondary pollutant ozone (O_3_). The main source of these pollutants is traffic and industry. As shown in recent review articles, there has been a rapid increase in the research investigating the effects of ambient air pollution on adverse birth outcomes [15]–[21]. Most studies use air pollutant data from large networks of fixed site monitors, combined with large retrospective birth cohorts obtained from government birth registries. Despite inconsistencies in the methods employed and the results reported, there is growing evidence suggesting that ambient air pollution during pregnancy is associated with adverse birth outcomes. However, there has been limited research on the effect of ambient air pollution during critical periods of pregnancy on congenital anomalies.

To date, there have only been four studies that focused on the effect of ambient air pollution on congenital anomalies, namely heart defects and cleft lip or palate. The first was conducted in Southern California where ambient CO during the second month of gestation was positively associated with an increased risk of ventricular septal defects [22]. A similar case–control study in Texas examined exposures during weeks 3–8 of gestation and reported positive associations between: ambient CO and multiple conotruncal defects and Tetralogy of Fallot, PM and isolated atrial septal defects, and SO_2_ and isolated ventricular septal defects [23]. A more recent study was conducted in Taiwan where ambient O_3_ during the first two months of pregnancy was positively associated with an increased risk of cleft lip (with or without cleft palate) [24]. A study in Atlanta, Georgia, examined exposures during weeks 3–7 of gestation and the risks of cardiovascular birth defects [25]. The study found only one statistically significant association, between PM_10_ and patent ductus.

Although ambient air pollution levels in Brisbane, Australia are reasonably low compared to many larger cities, previous research has shown that ambient air pollution in Brisbane has been associated with increased hospitalizations among children and the elderly [26], [27], increased risk of preterm birth [28], and reduced fetal growth [29]. Therefore the main aim this research was to examine potential associations between ambient air pollution in Brisbane and congenital anomalies, namely heart defects and cleft lip or palate, for comparison with previous research.

## Materials and Methods

### Study subjects and design

We used a population based case–control design and matched cases for each congenital defect with five controls in our retrospective birth cohort (1∶5 matching). We matched according to the following criteria: mother's age (±2 years), marital status (married /never married or de facto and other), indigenous status (yes/no), number of previous pregnancies, month of LMP (±1 month), area-level SES (based on deciles of an SES index), and distance to pollution monitor. We used matching in order to compare mothers of a similar age and social class. We matched on month of LMP to control for the effect of season on birth defects [30]–[33]. We matched on distance to monitor so that cases and controls had a similar degree of measurement error in air pollution.

Birth outcome data were collected from the Queensland Health Perinatal Data Collection Unit, which routinely collects data from all public and private hospitals in Brisbane together with data submitted voluntarily from homebirths. The data used in this study comprised all singleton births for the period of 1 January 1998 to 30 December 2004. Information was collected on the date of delivery, date of the last menstrual period (LMP), outcome of delivery (live born/stillborn), gestation (weeks), birth weight, a reported congenital anomaly (cardiac, cleft lip or palate defects), neonate gender, age of mother, first pregnancy (yes/no), marital status, indigenous status, and the statistical local area (SLA) the mother resided in at the time of delivery. In Brisbane most SLAs are smaller than postal areas and therefore the residential areas of the mothers are more refined.

For a measure of socio-economic status (SES) we linked an index of relative socio-economic disadvantage to each SLA. The index of relative socioeconomic disadvantage is an area level measure of SES developed by the Australian Bureau of Statistics and is derived from area attributes such as low income, low educational attainment, high unemployment, and jobs in relatively unskilled occupations, where a low score indicates socioeconomic disadvantage [34]. The index of relative socioeconomic disadvantage was categorized into deciles based on the SLAs within Queensland.

For comparison with previous research, we classified the cardiac defects into similar groupings used by Gilboa and colleagues [23]. In addition to cardiac defects, we examined cleft lip (with or without cleft palate). Table 1 shows the diagnostic groupings for all the defects analyzed.

**Table 1:** Classification of the birth defect groupings.

Diagnostic grouping	Selected birth defects
Aortic artery and valve defects	Aortic atresia, coarctation of the aorta, insufficiency of the aortic valve, aortic valve stenosis, interrupted aortic arch, hypoplasia of the aorta, persistent right aortic arch, overriding aorta, other aortic valve anomalies
Pulmonary artery and valve defects	Pulmonary atresia (valve or artery), pulmonary valve stenosis, insufficiency of the pulmonary valve, total anomalous pulmonary venous return, other pulmonary valve and artery anomalies (specified, unspecified)
Atrial septal defects	Atrial septal defect, single common atrium, other atrial septal defect (specified, unspecified)
Ventricular septal defects	Ventricular septal defect, other ventricular septal defect (specified, unspecified)
Conotruncal defects	Common truncus, transposition of the great vessels, other transposition of the great vessels (specified, unspecified), double outlet right ventricle, tetralogy of fallot
Endocardial cushion and mitral valve defects	Atrioventricular septal defect, hypoplastic left heart syndrome, mitral stenosis/insufficiency, endocardial cushion defects
Cleft lip	Cleft lip
Cleft lip/palate	Cleft lip with cleft palate
Cleft palate (isolated)	Cleft palate

### Exposure assessment

**Figure 1:**
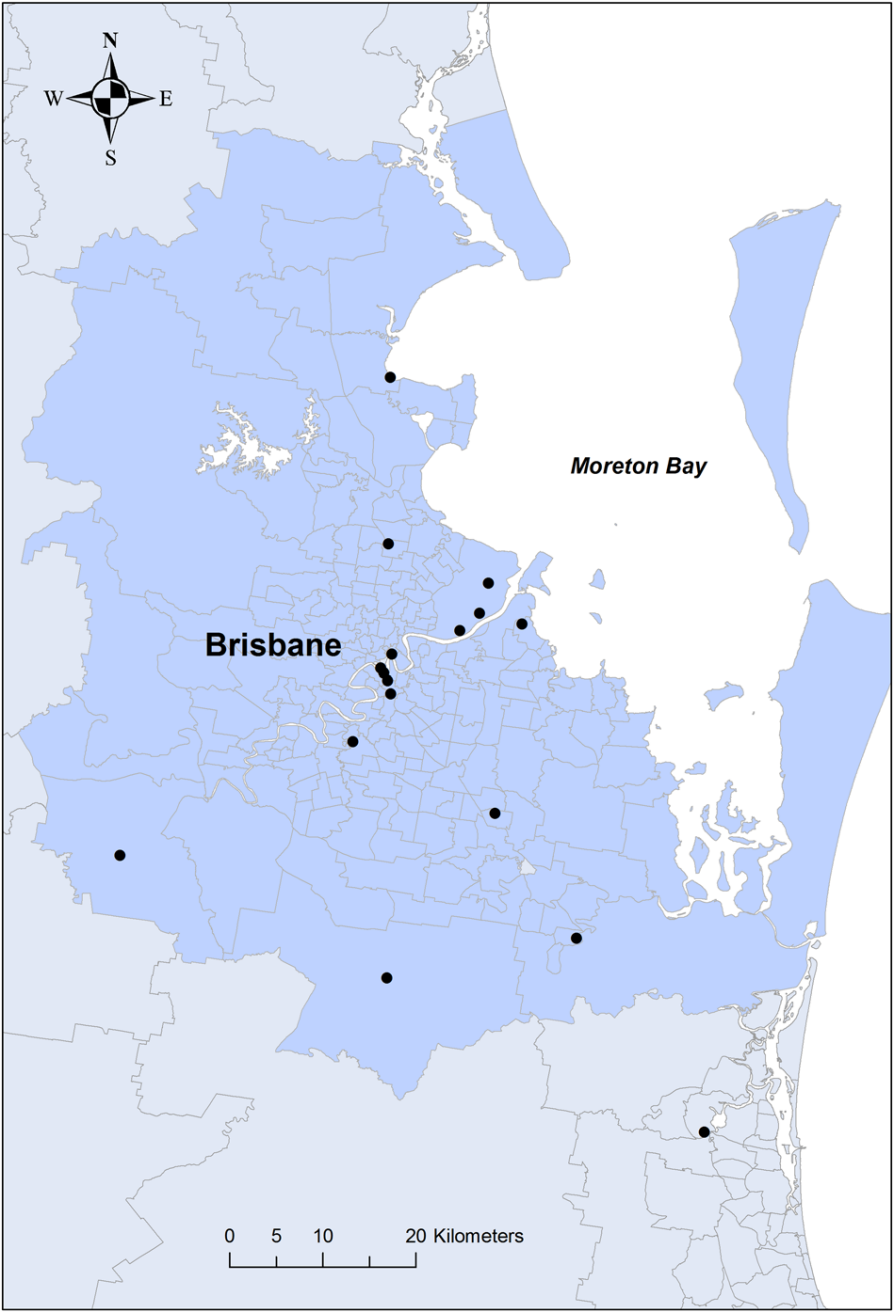
Geographic area of the birth cohort (shaded area) and location of 18 fixed air pollution monitors (black dots) in the Brisbane area. The borders represent statistical local areas (SLAs).

For the period January 1997 to December 2004, air pollution data for Brisbane and surrounding areas were obtained from the Air Services Unit, Queensland Environmental Protection Agency. Air quality was monitored at 18 different fixed sites with the majority located within a 30-kilometer radius of Brisbane city (Figure 1). Hourly readings were obtained for O_3_ (reported as parts per billion [ppb]), NO_2_ (reported as ppb), SO_2_ (reported as ppb), CO (reported as part per million [ppm]), and particulate matter with an aerodynamic diameter <10 µm (PM_10_ reported as micrograms per cubic meter). A daily average was calculated for PM_10_, NO_2_, and SO_2_, whereas an 8-hour average was calculated for CO and O_3_. Not all pollutants were monitored at all 18 sites for the entire study period.

Similar to our previous research in Brisbane [29], for the exposure assessment we took the following steps to assign air pollution exposures to each mother/neonate pair. We obtained the digital boundaries of the Queensland SLAs from the Australian Bureau of Statistics [35] and calculated the distance from the centroid of each SLA to each monitoring site. Based on the mothers' SLA, we assigned an estimate for each air pollutant on each day of gestation using the closest monitoring site. If there were missing data from the closest site for a particular day of gestation, then the reading was taken from the next closest site without missing data. If the daily readings were missing across all sites, then the daily exposure estimate was left as missing.

We then calculated average exposure estimates over the days of gestation for weeks 3–8 of gestation (post LMP) as this is the critical period of gestation associated with congenital anomalies [36]. This average was based on 42 days.

By using information from individual monitoring sites we hoped to exploit the spatial variation in air pollution to look for differences in risk. By using a relatively short exposure period the study also used the temporal variation in air pollution. Week-to-week variations in pollutants in Brisbane are caused by many factors including the number of cars on the roads (which decrease during school holidays) and bushfires.

### Data analysis

We used conditional logistic regression to examine the differences in pollution exposure between cases and matched controls. All models adjusted for neonate gender. The odds ratios (ORs) are shown for an interquartile range (IQR) increase in air pollutant. Air pollutants were entered into the model as continuous covariates. An IQR increase can be thought of as the difference between a moderately good and a moderately bad exposure period. This makes the changes seen with different air pollutants more comparable.

Women who lived closer to an air pollution monitor should have more accurate estimates of their exposure compared with women who lived further away. Measurement error in air pollution would bias any association towards the null, so to quantify this bias we ran sensitivity analyses using only women who lived within 6 and 12 km of a monitor [29]. These distances might seem quite large for accurate pollutions assessment, however there were very few cases within 2 km of a monitor (<0.1% of the sample) and so using this exclusion would drastically reduce the statistical power of the study.

To assess any bias from over-matching, we compare the results from the matched analyses to additional analyses that used no matching, and instead randomly matched five controls to each case.

All models were fitted using a Bayesian paradigm, using vague priors for all unknown parameters. We used a vague Normal prior with zero mean and variance of 1000 for all regression parameters, and a gamma prior with a shape an inverse scale parameter of 0.001for all inverse-variance parameters. We used the JAGS software to estimate the parameters [37]. We used a burn-in of 5,000 MCMC iterations and a sample of 5,000. We checked the convergence of the chains using the “coda” library in the R software package.

## Results

Descriptive statistics for pollution levels during the study period are shown in Table 2. The most complete data for the study period were for NO_2_ and O_3_, which were monitored at most sites, whereas the least complete data were for CO, which was monitored at only 4 of the 18 sites.

**Table 2:** Daily air pollution levels in Brisbane (January 1998 to December 2004).

	PM_10_ (µg/m^3^)	NO_2_ (ppb)	O_3_ (ppb)	SO_2_ (ppb)	CO (ppm)
Number of monitoring sites	11	16	15	7	4
Days missing data across all sites	1	1	1	11	400
Mean (min, max)^a^					
All seasons	18.0 (4.4, 151.7)	8.2 (1.4, 22.7)	25.8 (4.3, 54.4)	1.5 (0, 7.1)	1.1 (0.02, 7.0)
Summer	18.1	5.2	24.8	1.5	0.7
Autumn	15.8	8.3	23.0	1.6	1.1
Winter	17.5	11.3	24.2	1.4	1.5
Spring	20.7	7.9	31.1	1.4	0.9

a based on an average across all available sites.

PM_10_, NO_2_, SO_2_ = 24 hour average; O_3_, CO = 8 hour average.

Table 3 shows descriptive statistics on the birth cohort. There were 150,308 births during the study period. The birth defect with the highest rate was ventricular septal defects (14.7 per 10,000 births), followed by atrial septal defects (8.4 per 10,000 births).

**Table 3:** Characteristics of the study subjects (n = 150, 308).

Variable, statistics	Statistics
Mother's indigenous status, n (%)	
Yes	3347 (2.2)
No	146,961 (97.8)
Mother's marital status, n (%)	
Married or de facto	131,114 (87.2)
Never Married	16,989 (11.3)
Other	2205 (1.5)
Mother's age (years), mean (SD)	29.0 (5.6)
Number of previous pregnancies, median (IQR)	1 (0–2)

Table 4 shows the odd ratios for the risk of specific congenital cardiac defects associated with ambient air pollution averaged over weeks 3–8 of pregnancy. When analyzing all births there was no indication that ambient air pollution in Brisbane was associated with a higher risk of cardiac defects. In fact, the only statistically significant results suggested that ambient CO was inversely associated with ventricular septal defects and conotruncal defects. There were also no adverse effects found when restricting the analyses to only include births where the mother resided within 12 kilometers of an air monitoring station. However, among births within 6 kilometers of a monitor, a 5 ppb increase in O_3_ was associated with an increased risk of pulmonary artery and valve defects (OR 2.96, 95% CI: 1.34, 7.52) while a 0.6 ppb increase in SO_2_ was associated with an increased risk of aortic artery and valve defects (OR 10.76, 95% CI: 1.50, 179.8). Results from the unmatched analyses still showed CO to be inversely associated with ventricular septal defects, while PM_10_ was now adversely associated with ventricular septal defects.

**Table 4:** Adjusted odds ratios (95% credible intervals) for the risk of specific congenital cardiac defects associated with ambient air pollution averaged over weeks 3–8 of pregnancy.

Number of cases	Aortic artery and valve defects	Atrial septal defects	Pulmonary artery and valve defects	Ventricular septal defects	Conotruncal defects	Endocardial cushion and mitral valve defects
	63	127	64	222	63	33
***Matched results***						
**All births**						
PM_10_	1.10 (0.76, 1.56)	1.06 (0.86, 1.30)	0.90 (0.61, 1.29)	0.87 (0.73, 1.04)	0.80 (0.54, 1.19)	1.29 (0.82, 2.04)
NO_2_	1.20 (0.70, 2.08)	1.09 (0.78, 1.55)	1.04 (0.61, 1.76)	0.90 (0.67, 1.19)	0.62 (0.34, 1.12)	1.56 (0.75, 3.12)
O_3_	1.05 (0.73, 1.50)	1.03 (0.77, 1.37)	0.93 (0.61, 1.34)	0.82 (0.66, 1.01)	0.98 (0.67, 1.43)	0.83 (0.49, 1.44)
SO_2_	0.87 (0.61, 1.21)	1.30 (0.99, 1.74)	0.93 (0.65, 1.31)	0.84 (0.69, 1.02)	0.71 (0.48, 1.07)	0.86 (0.52, 1.45)
CO	0.85 (0.49, 1.49)	0.83 (0.62, 1.12)	0.64 (0.32, 1.21)	0.61 (0.47, 0.78)	0.38 (0.18, 0.74)	0.61 (0.31, 1.14)
**Births with ≤12 km average distance to monitor**						
PM_10_	1.83 (1.16, 2.98)	1.07 (0.84, 1.37)	0.69 (0.43, 1.08)	0.85 (0.69, 1.03)	0.94 (0.55, 1.49)	1.28 (0.75, 2.19)
NO_2_	1.17 (0.64, 2.20)	1.04 (0.71, 1.51)	1.06 (0.62, 1.92)	0.86 (0.62, 1.18)	0.63 (0.35, 1.14)	1.58 (0.70, 3.74)
O_3_	1.30 (0.88, 1.96)	1.11 (0.82, 1.49)	0.91 (0.61, 1.34)	0.94 (0.74, 1.18)	1.05 (0.70, 1.59)	0.98 (0.54, 1.79)
SO_2_	1.42 (0.73, 2.85)	1.23 (0.85, 1.79)	1.12 (0.65, 1.91)	0.85 (0.63, 1.14)	0.58 (0.30, 1.04)	1.20 (0.57, 2.65)
CO	0.75 (0.29, 1.84)	0.92 (0.57, 1.48)	0.50 (0.17, 1.27)	0.61 (0.41, 0.91)	1.09 (0.32, 3.97)	0.83 (0.27, 2.17)
**Births with ≤6 km average distance to monitor**						
PM_10_	1.43 (0.73, 2.90)	0.88 (0.60, 1.27)	1.46 (0.76, 2.73)	0.90 (0.68, 1.18)	0.66 (0.27, 1.45)	0.90 (0.44, 1.86)
NO_2_	0.89 (0.32, 2.41)	1.15 (0.61, 2.20)	1.26 (0.48, 3.22)	0.80 (0.52, 1.22)	0.88 (0.33, 2.40)	6.93 (0.93, 114.81)
O_3_	1.76 (0.96, 3.34)	0.76 (0.46, 1.21)	2.96 (1.34, 7.52)	1.37 (0.99, 1.93)	1.04 (0.51, 2.17)	0.75 (0.03, 15.78)
SO_2_	10.76 (1.50, 179.83)	1.61 (0.84, 3.08)	0.70 (0.16, 1.96)	0.64 (0.35, 1.08)	0.27 (0.07, 0.81)	—
CO	1.27 (0.16, 8.07)	0.87 (0.39, 1.95)	1.25 (0.12, 15.27)	0.70 (0.35, 1.30)	—	—
***Unmatched results (5 randomly selected controls, no restriction on distance to monitor for cases)***						
**All births**						
PM_10_	1.09 (0.84, 1.39)	1.14 (0.98, 1.33)	0.99 (0.78, 1.24)	1.15 (1.02, 1.30)	0.97 (0.74, 1.24)	0.94 (0.68, 1.26)
NO_2_	1.03 (0.80, 1.32)	1.08 (0.90, 1.28)	0.96 (0.75, 1.23)	1.02 (0.89, 1.17)	1.02 (0.79, 1.30)	1.34 (0.94, 1.92)
O_3_	0.90 (0.70, 1.13)	0.99 (0.84, 1.18)	1.09 (0.86, 1.40)	0.93 (0.81, 1.05)	0.87 (0.68, 1.12)	1.03 (0.74, 1.41)
SO_2_	0.83 (0.60, 1.13)	0.84 (0.69, 1.01)	0.82 (0.60, 1.08)	0.87 (0.74, 1.01)	0.83 (0.61, 1.12)	0.91 (0.60, 1.40)
CO	0.80 (0.60, 1.08)	0.83 (0.69, 1.01)	1.02 (0.75, 1.38)	0.68 (0.58, 0.78)	0.88 (0.65, 1.19)	0.79 (0.53, 1.17)

Adjusted for: Neonate sex.

Unit increases for each pollutant: PM_10_ = 4 µg/m^3^, NO_2_ = 4 ppb, O_3_ = 5 ppb, SO_2_ = 0.6 ppb, CO = 0.6 ppm.

Table 5 shows the odds ratios for the risk of cleft lip/palate associated with ambient air pollution averaged over weeks 3–8 of pregnancy. Similar to the cardiac defects, mixed results were found. The only statistically significant results came from analyses that included all births regardless of the average distance to a monitor. The only adverse association was between SO_2_ and cleft lip with or without cleft palate (OR 1.27, 95% CI: 1.01, 1.62). Inverse associations were found between PM_10_ and cleft palate (OR 0.69, 95% CI: 0.50, 0.93), and CO and cleft lip with or without cleft palate (OR 0.59, 95% CI: 0.42, 0.80). None of the statistically significant effects were present in the unmatched analyses.

**Table 5:** Adjusted odds ratios (95% credible intervals) for the risk of congenital cleft lip/palate associated with ambient air pollution averaged over weeks 3–8 of pregnancy.

Pollutant	Cleft lip	Cleft palate	Cleft lip with or without cleft palate
Number of cases	57	100	145
***Matched results***			
**All births**			
PM_10_	1.05 (0.72, 1.51)	0.69 (0.50, 0.93)	1.05 (0.84, 1.30)
NO_2_	1.24 (0.66, 2.32)	0.73 (0.46, 1.15)	1.21 (0.84, 1.75)
O_3_	0.91 (0.58, 1.42)	0.87 (0.62, 1.20)	0.82 (0.63, 1.06)
SO_2_	1.40 (0.96, 2.01)	0.84 (0.64, 1.09)	1.27 (1.01, 1.62)
CO	0.71 (0.41, 1.25)	0.74 (0.49, 1.10)	0.59 (0.42, 0.80)
**Births with ≤12 km average distance to monitor**			
PM_10_	1.16 (0.72, 1.82)	0.53 (0.29, 0.87)	1.03 (0.79, 1.34)
NO_2_	1.24 (0.63, 2.53)	0.81 (0.34, 1.75)	1.25 (0.82, 1.89)
O_3_	1.06 (0.65, 1.73)	0.78 (0.44, 1.36)	0.88 (0.65, 1.17)
SO_2_	1.22 (0.64, 2.22)	0.62 (0.22, 1.60)	1.05 (0.74, 1.46)
CO	0.92 (0.43, 2.00)	1.60 (0.46, 5.69)	0.60 (0.36, 0.96)
**Births with ≤6 km average distance to monitor**			
PM_10_	1.03 (0.56, 1.82)	0.71 (0.49, 1.00)	0.83 (0.58, 1.19)
NO_2_	0.89 (0.31, 2.52)	0.70 (0.41, 1.16)	1.08 (0.59, 1.92)
O_3_	0.83 (0.40, 1.67)	0.88 (0.62, 1.23)	0.71 (0.45, 1.09)
SO_2_	1.24 (0.37, 4.41)	0.79 (0.51, 1.21)	0.90 (0.46, 1.76)
CO	1.51 (0.31, 6.29)	1.17 (0.62, 2.21)	0.49 (0.20, 1.19)
***Unmatched results (5 randomly selected controls, no restriction on distance to monitor for cases)***			
**All births**			
PM_10_	1.01 (0.79, 1.27)	0.89 (0.72, 1.10)	1.04 (0.89, 1.21)
NO_2_	0.96 (0.73, 1.24)	0.86 (0.69, 1.08)	0.95 (0.79, 1.14)
O_3_	0.98 (0.75, 1.28)	0.85 (0.69, 1.04)	1.01 (0.86, 1.19)
SO_2_	1.05 (0.79, 1.39)	0.90 (0.72, 1.12)	1.07 (0.88, 1.28)
CO	1.07 (0.78, 1.48)	0.95 (0.77, 1.17)	0.92 (0.76, 1.11)

Adjusted for: Neonate sex.

Unit increases for each pollutant: PM_10_ = 4 µg/m^3^, NO_2_ = 4 ppb, O_3_ = 5 ppb, SO_2_ = 0.6 ppb, CO = 0.6 ppm.

## Discussion

This study investigated the possible association between ambient air pollution and the risk of specific birth defects, namely cardiac and cleft lip/palate. We found mixed results across all analyses and no consistent patterns were observed with regard to adverse effects and distance to a monitor. Although we did find several statistically significant adverse associations, there were also significant inverse associations. Therefore the few adverse effects need to be interpreted with caution. Also, given the number of analyses performed, there is a possibility that the statistically significant associations occurred by chance.

### Comparisons with previous studies

There has also been inconsistency across the four previous studies that have examined associations between ambient air pollution and birth defects. Also, each of these studies found only one or two significant associations among a large number of analyses. For example, in Southern California [22], exposure to ambient CO, NO_2_, O_3_ and PM_10_ during each of the first three months of pregnancy was examined and results showed that CO during month two was associated with an increased risk of cardiac ventricular septal defects with an exposure-response pattern exhibited across the CO quartiles of exposure (OR 2.95, 95% CI: 1.44, 6.05 for the highest quartile [≥2.39 ppm] exposure group compared to the lowest [<1.14 ppm]). The only other pollutant associated with a defect was O_3_ during month two, which was associated with an increased risk of aortic artery and valve defects (OR 2.68, 95% CI: 1.19, 6.05 for the highest quartile of exposure). Whereas in the Texas study [23] CO and O_3_ were not adversely associated with ventricular septal defects or aortic artery and valve defects, respectively. Also this study showed an inverse association between CO and ventricular septal defects. The main results from the Texas study [23] showed that CO was associated with multiple conotruncal defects (OR 1.46, 95% CI: 1.03, 2.08 for the highest quartile of exposure [≥0.7 ppm] compared to the lowest [<0.4 ppm]), and Tetralogy of Fallot (OR 2.04, 95% CI: 1.26, 3.29 for the highest quartile of exposure), PM_10_ was associated with atrial septal defects (OR 2.27, 95% CI: 1.43, 3.60 for the highest quartile of exposure [≥29 µg/m^3^]), and SO_2_ was associated with ventricular septal defects (OR 2.16, 95% CI: 1.51, 3.09 for the highest quartile of exposure [≥2.7 ppb]). The very latest study based in Atlanta, Georgia, examined 12 types of cardiovascular birth defect and five pollutants but found only one statistically significant association: between PM_10_ and patient ductus [25]. Once again, our results are not consistent with any of the Texas [23], Southern California [22] or Georgia [25] study results.

With regard to oral clefts, namely cleft lip with or without palate (CL/P), the latest of the previous studies that focused on this outcome was a case–control study that examined maternal exposure to various air pollutants during the first three months of pregnancy. Based on spatially interpolated data from all fixed monitoring sites across Taiwan, exposure estimates for PM_10_, SO_2_, NOx, O_3_, and CO were averaged over each of the first three months of pregnancy. Interestingly, of all the pollutants examined, only O_3_ during the first two months of pregnancy was significantly associated with an increased risk of CL/P (OR 1.17, 95% CI: 1.01, 1.36; OR 1.22, 95% CI: 1.03, 1.46 per 10 ppb increase respectively) [24]. However, in Southern California, Texas, and the current study, O_3_ was not statistically associated with an increased risk of cleft lip/palate.

Our results for cleft lip/palate showed that SO_2_ was associated with CL/P when using all births, but this adverse effect disappeared when only using births within 12 and 6 km from a monitor. We would have expected any association to become stronger when using data closer to the monitor as the accuracy of the exposure increases, but in this case the association became weaker. Also, similar to results for cardiac defects, inverse associations were also found for cleft lip/palate.

The results in the current study are inconsistent with our previous research that found ambient air pollution in Brisbane to be associated with an increased risk of preterm birth [28], and reduced fetal growth [29]. One explanation is that because birth defects are rare, and the timing of the environmental insult is very precise for particular defects, the methods employed in this study were not sensitive enough to detect a possible, and consistent, association between air pollution and the defects examined. Whereas, for fetal growth and preterm birth the exact timing of exposure may not need to be as precise for an effect to be detected as the adverse effect occurs over a longer time period. Of course another explanation is that although air pollution may decrease birth size it is not a cause of birth defects.

The design of this study was similar to previous studies, although one potentially important difference is that we estimated the effect of air pollution as a continuous exposure (as did [24], [25]), whereas other studies categorized exposure into quartiles [22], [23]. Using a continuous exposure will give more statistical power if a log-linear association exists between exposure and risk of a defect. Breaking the exposure into quartiles reduces power, but puts no restrictions on the shape of the exposure-risk relationship. We preferred to keep exposure as continuous because: a) it is biologically plausible that increased exposure leads to a steadily increasing risk which would be captured by a log-linear curve, b) using groups will only give better results when the cut-points are selected to break the exposure into substantively different exposure levels. Using quartiles makes this decision easier (and standardizes exposures across different pollutants), but the choice is also rather arbitrary, and should be justified against other cut-points (such as tertiles or quintiles).

### Measurement error in air pollution

It is possible that air pollution is associated with birth defects, but that the association was too small to detect using this sample. Another possibility is that the measurement error in pollution exposure was too great. Using an ambient network of pollution monitors to assess individual exposure introduces measurement error because of the distance between the monitor and the subject, and the individual modifiers of exposure such as air conditioning. Studies using network pollution data in Brisbane have previously found significant effects of air pollutants on fetal size and hospitalizations [26]–[29], but these studies were based on a larger sample size. Birth defects are thankfully rare, but this means that the power to detect a difference in observational studies can be low. For example, to detect a linear increase in the excess risk of a defect of 0.1 for every IQR increase in ozone would require 1527 cases (based on 5 controls per case, an 80% power, a 5% significance level and the observed distribution of ozone exposure) [38]. Detecting an increase in excess risk of 0.05 would require 4246 cases. The largest number of defects in this study was 222 for ventricular septal defects (despite the study containing 150,308 births), therefore this study has a low power to detect small increases in risk.

Power may also be improved by using more personal measures of exposure. The ideal solution is to put pollution monitors in a cohort of pregnant mothers, but this would be very expensive. A cheaper alternative is to measure the road network surrounding the mother's home using their geocoded address, as a proxy measure of pollution. In Brisbane, 70–80% of air pollution comes from traffic, so these measures are likely to be better measures of exposure to air pollution for those women who live far from a monitoring site. Studies using these proxy measures have shown associations between increased road density or proximity to major roads and low birth weight and preterm births [39].

Our results showed some inverse associations between defects and exposure to PM_10_, SO_2_ and CO. These effects are difficult to interpret and may just be type I errors. From the matched results there were 8 inverse associations from a total of 42 tests (Tables 4–5), this represents 20% of tests which is much higher than the 5% we would expect. We were concerned that these differences may have been caused by over-matching, so we also created estimates based on 5 randomly selected controls without matching. These results also showed inverse associations for CO. This result leads us to believe that the cases of some birth defects have lower than average CO levels. In south-east Queensland around 83% of carbon monoxide exposure is from motor vehicles [40]. So low exposure to CO is a marker of low exposure to traffic, which will be more common in semi-rural areas. Exposure to pesticide is a known cause of birth defects [41], and this exposure may be more common in semi-rural areas due to agricultural activity. A land use regression analysis would be useful to quantify this risk [18], however such an analysis would need each mother's actual address rather than just their postcode.

Some of the non-statistically significant results are worthy of comment. The associations between NO_2_ and endocardial cushion and mitral valve defects (Table 4) always had a positive odds ratio and a lower credible interval relatively close to 1. This is suggestive that a true association may exist but was not statistically significant in this sample. One reason for a lack of statistical significance may be a lack of power, and this type of defect had the smallest number of cases (33) and hence the lowest power to detect any association.

### Limitations

This study does have some limitations that are common in this field of research. The exposure is based on data from the closest monitoring site to the mothers' residence at the time of birth and therefore residential mobility during pregnancy may have occurred. Studies have shown that approximately 12–33% of women move address during pregnancy [42]–[45]. Potential exposure misclassification due to residential mobility is usually nondifferential and therefore will weaken any true association [42], hence we may have missed some true associations. Avoiding this bias is only possible with more detailed study designs. Information on various potential confounders such as maternal smoking, drug and alcohol use, diet, and occupational exposures, was unavailable. However, many of these factors are constant over time and will not be confounded with the week-to-week changes in ambient air pollution levels. Differences between these factors at an area level would have been partly controlled by using area-level SES.

### Summary

This study found mixed results and it is difficult to conclude whether ambient air pollution in Brisbane has an adverse association with the birth defects examined. Results from these studies need to be interpreted with caution and improvement in exposure assessment is needed before unequivocal conclusions can be reached.
